# Immunomodulatory effects of epiphytic *Loranthus micranthus* leaf extracts collected from two host plants: *Psidium guajava* and *Parkia biglobosa*

**DOI:** 10.1186/s12906-023-04282-4

**Published:** 2024-01-02

**Authors:** Ngozi Dorathy Idoko, Ifeoma Felicia Chukwuma, Florence Nkechi Nworah, Sopuruchukwu Elizabeth Mba, Parker Elijah Joshua, Okwesilieze Fred Chiletugo Nwodo, Wafaa Fouzi Abusudah, Najlaa Hamed Almohmadi, Michel de Waard

**Affiliations:** 1 Department of Biochemistry, Faculty of Biological Sciences, University of Nigeria, Nsukka, Nigeria; 2 Department of Medical Biochemistry, Faculty of Basic Medical Sciences, College of Medicine Enugu State University of Science and Technology, Enugu, Nigeria; 3 Clinical Nutrition Department, College of Applied Medical Sciences, UMM AL-QURA University, Makkah, 24381 Saudi Arabia; 4 Smartox Biotechnology, 6 rue des Platanes, 38120, Saint-Egrève, France; 5 L’institut du thorax, INSERM, CNRS, Univ nantes, F-44007 Nantes, France; 6 Université de Nice Sophia-Antipolis, LabEx «Ion Channels, Science & Therapeutics, F-06560 Valbonne, France

**Keywords:** Antibodies, Cell-mediated immunity, Humoral immunity, Immunomodulatory, *Loranthus micranthus*, Phytochemicals

## Abstract

**Background:**

Immunological abnormalities are implicated in the pathogenesis of many chronic diseases. Due to the drug-related adverse effects of currently available orthodox immunomodulators, natural immunomodulators are being looked upon as potential agents to replace them in therapeutic regimens. This research aimed to investigate the immunomodulatory potential of *L. micranthus* extracts epiphytic on *Psidium guajava* (LMPGE) and *Parkia biglobosa* (LMPBE)*.*

**Methods:**

Phytochemical screening and acute toxicity testing were carried out to identify the phytoconstituents and safety profiles of the extracts. The extracts’ innate and adaptive immunomodulatory potentials were determined in experimental animals using in vivo leucocyte mobilization, delayed-type hypersensitivity (DTH) response, hemagglutination antibody titre, and cyclophosphamide-induced myelosuppression models. Levamisole was used as the standard drug throughout the study.

**Results:**

Compared to LMPBE, LMPGE contained significantly (*p* <  0.05) more tannins, cyanogenic glycosides, saponins, reducing sugars, glycosides, flavonoids, and alkaloids. Furthermore, the groups treated with the extracts had a significant (p <  0.05) increase in the total number of leucocytes, neutrophils, basophils, and antibody titers relative to the untreated control. In the same way, the treatment raised TLC in cyclophosphamide-intoxicated rats, with 250 mg/kg b. w. of LMPGE and LMPBE recording 9712.50 ± 178.00 and 8000.00 ± 105.00 ×  109 /L, respectively, compared to 3425.00 ± 2 5.00 × 109 /L in the untreated group. Overall, LMPGE was more effective.

**Conclusions:**

The findings from this study suggest that *L. micranthus* epiphytic in *Psidium guajava* and *Parkia biglobosa* has possible immune stimulating potential.

## Background

The past decade has experienced a rise in the development of several immunomodulators, which are primarily used as immunoadjuvants to enhance vaccine efficacy; immunostimulants to induce or stimulate immune mediators and cellular components of immune responses for the treatment of infectious diseases and immunodeficiency disorders such as AIDS; immunosuppressants to suppress immunological responses after therapy for autoimmune diseases, hypersensitivity reactions, and organ transplants [1]. Various synthetic and recombinant drugs, including levamisole, pentoxifylline, thalidomide, and isoprenaline, are immunomodulators prescribed in clinical settings [2]. Administration of levamisole enhances the maturation of thymocytes, improves DTH in immunosuppressed patients, and restores T-suppressor and T-helper cell activity [3].

However, the adverse side effects of levamisole, which include but are not limited to right heart failure, pulmonary hypertension, vasculitis, inflammatory leukoencephalopathy, agranulocytosis, retiform purpura, arthralgia, vasculopathy, and leukoencephalopathy [4–6], limit their intended use. Consequently, immunomodulatory agents with better safety and therapeutic effectiveness are still needed [1]. The increased shift in research attention from orthodox medicine to alternative herbal systems is due to their high safety profile, accessibility, therapeutic efficacy, and multi-targeted approach [7, 8]. According to a WHO report, more than 70% of the population in developing countries rely on medicinal plants due to their better safety profile and improved efficacy [9]. In addition, the high cost of orthodox immune modulators limits their effective usage by most patients, leading to a high morbidity and mortality rate from immune-mediated diseases. The only hope for such patients is the discovery of natural agents within their environment, which will have little or no cost. Interestingly, research on medicinal plants will provide valuable entities for developing immunomodulatory agents to supplement or replace current chemotherapy [10, 11]. Previous studies demonstrated that phytochemicals derived from plants, such as tannins, alkaloids, glycosides, polysaccharides, triterpenoids, and flavonoids, have immunomodulatory properties [12, 13]. Several plants contain these phytochemicals, including African mistletoe (*Loranthus micranthus)* [14, 15].

*Loranthus micranthus,* an obligate semi-parasitic plant of the family *Loranthaceae*, is native to Nigeria, America, Europe, and other parts of Africa [14]. It grows on various host trees and shrubs, including *Azadirachta indica, Kola acuminata, Pentaclethra macrophylla,* and *Persia americana* [16]*.* Nevertheless, it has been spotted on other trees, including *Psidium guajava* (known as “*guaba”* in Yoruba, “*goba*” in Hausa, and “*gova”* in Igbo) and *Parkia biglobosa* (called “*Ugba*” in Igbo, “*dawadawa*” in Hausa, and “*Iru”* in Yoruba). *L. micranthus* has a strong reputation in traditional medicine for its use in treating various ailments, notably high blood pressure, diabetes, cardiovascular illnesses, and conditions affecting the human immune system, as well as headaches, epilepsy, infertility, agglutination, menopausal syndrome, and rheumatism [17]. The pharmacological properties reported in the plant are not limited to antidiabetic, antihypertensive, hypolipidemic, and antimicrobial activities [16, 18]. Leveraging on the high ethnomedicinal and pharmaceutical actions of mistletoe and the paucity of information on the comparative immunomodulatory activity of *L. micranthus* epiphytic on *P. guajava and P. biglobosa*, which are known to have numerous established health benefits. This study investigated the immunomodulatory effects of *L. micranthus* leaves collected from *P. guajava and P. biglobosa* on rats’ innate and adaptive immune responses and cyclophosphamide-induced myelosuppression.

## Methods

### Plant collection and identification

The plant material (*L. micranthus* leaves) was collected from Orba in Nsukka Local Government Area, Nigeria, from different wild host trees, *P. guajava* and *P. biglobosa.* Plant taxonomist Alfred Ozioko identified and collected the leaves. The voucher specimen of the leaves was registered in the herbarium of the International Centre for Ethnomedicine and Drug Development (InterCEDD) Nsukka, Enugu State, Nigeria (voucher number INTERCEDD/1603).

### Experimental animals

The experimental animals (160 Wistar albino rats and 36 Swiss mice) used for this research were procured from the same source. They were kept in stainless steel cages (five animals per cage) under the same environmental conditions to eliminate any confounding factors that might influence the experiment’s outcome. The animals were fed pelletized feed (Vital Feed Nig. Ltd.) and tap water ad libitum. Standard laboratory conditions and protocols recommended by the National Institute of Health Guidelines for the Use and Care of Laboratory Animals (revised 1985, Pub No. 85–23) (1985) were strictly followed throughout the experiment. After the investigation, the death of the animals was humanely induced with an intraperitoneal injection of 200 mg/kg of sodium pentobarbital following the American Veterinary Medical Association (AVMA) guidelines [19].

### Antigen preparation

The antigen was prepared from fresh sheep red blood cells (SRBCs). The SRBC was washed three times in pyrogen-free, sterile normal saline by centrifugation for 10 min at 3000 x g rpm. Then, the concentration of the washed SRBCs was adjusted to 109 cells/mL with normal saline by counting the cells using a Neubauer chamber in a light microscope [20]. After that, concentrated SRBC was used for the challenge and immunization.

### Drugs

The drugs employed as a standard immunostimulant and immunosuppressant were levamisole (25 mg/kg), procured from ICI Pakistan, and cyclophosphamide (25 mg/kg), sourced from Cadila Healthcare Limited, Germany, respectively. In addition, sheep red blood cells (SRBCs) were used as antigens for immunization and challenge.

### Preparation and extraction of the plant material

*L. micranthus* leaves were washed and air-dried for 6 days after being obtained from *P. guajava* and *P. biglobosa.* Then, they were pulverized in a laboratory grinder, and 200 g of each leaf was extracted for 72 h in a conical flask with 2.5 L of methanol (Sigma Cat. No. 107018) and distilled water in a ratio of 80:20. The methanol was diluted to increase the polarity, which is suitable for the extraction of phytochemicals present in the leaves [21, 22]. The macerate was filtered through Whatman 1 filter paper (paw size 11 m) and concentrated at 45 °C under reduced pressure (≤ 10 10 mmHg) using a rotary evaporator (Model Modulyo 4 k, Edward England) to obtain extracts of *L. micranthus* epiphytic on *P. guajava* and *P. biglobosa* designated as LMPGE and LMPBE, respectively.

### Qualitative phytochemical analysis

Harborne [23] and Trease and Evans [24] experimental procedures were used to screen the extracts for phytochemicals as follows:

**Steroids**: 2 mL of chloroform (Sigma Cat. No. 107024) and 1 mL of sulfuric acid (BDH Cat. No. BDH3072) were added to 0.1 g of each extract. The formation of a reddish-brown ring at the interface indicated the presence of steroids.

**Tannins:** A quantity of 0.1 g of each extract was added to deionized water (10 mL) and filtered before adding 3 drops of ferric chloride (BDH Cat. No. BDH7318). The formation of a greenish-brown precipitate (ppt) indicated the presence of tannins.

**Soluble Carbohydrate:** Each extract (0.1 g) was separately boiled with 2 mL of distilled water and filtered. Subsequently, a few drops of Molisch’s reagent were added to the filtrate. Conc. sulfuric acid was then gently poured down the side of the test tube to form a lower layer. A purple interfacial ring indicated the presence of carbohydrates.

**Cyanogenic glycosides:** A few drops of ferric chloride (5%) and 2 mL glacial acetic acid (Sigma Cat. No. 100066) were added to 0.1 g of each extract. The formation of the brown ring at the interface after adding 1 mL of conc. Sulfuric acid confirmed the presence of cyanogenic glycosides.

**Saponins:** Each extract (0.1 g) was added to deionized water (200 mL) and shaken vigorously for 15 min. The formation of the foam layer indicated the presence of saponins.

**Reducing Sugar:** Five milliliters (5 mL) of a mixture of equal parts of Fehling’s solution A (Sigma Cat. No. 89474) and B (Sigma Cat. No. 12603) were added to 0.5 g of each extract and then heated in a water bath for 5 min. Brick-red precipitate showed the presence of reducing sugar.

**Glycosides:** Dilute sulfuric acid (5 mL) was added to 0.1 g of each extract in a test tube, which was later boiled for 15 min in a water bath. The test tube was cooled and neutralized with a 20% potassium hydroxide solution (Sigma Cat. No. BDH9262). Then, 10 mL of a mixture of equal parts of Fehling’s solutions A and B was added and boiled for 5 min. A more dense brick-red precipitate indicated the presence of glycoside.

**Flavonoids:** The presence of flavonoids in each extract was determined by boiling 0.1 g of the extract in 10 mL of ethyl acetate (Sigma Cat. No. BDH1123) for 3 min. Then, 4 mL of the cooled filtrate was shaken with 1 mL of dilute ammonia solution (Sigma Cat. No. BDH7615). An intense yellow coloration confirmed the presence of flavonoids.

**Alkaloids:** Alkaloids were qualified by boiling 0.5 g of each extract with 6 mL of 1% aqueous HCl (Sigma Cat. No. 113136). A reddish-brown ppt formed after adding 2 drops of Wagner’s reagent to the filtrate indicated the presence of alkaloids.

**Terpenoids:** Chloroform (2 mL) was added to 0.1 g of each extract in a test tube. The formation of a reddish-brown color at the interface after adding conc. Sulfuric acid (1.5 mL) indicated the presence of terpenoids.

**Resin:** Acetic anhydride solution and 1 mL of conc. Sulfuric acid were added to 1 g of each extract. There was no formation of orange-to-yellow coloration to indicate the presence of resin.

**Fats and oils**: A small quantity of the leaf extract was pressed between two filter papers. There was no oil stain on the filter paper to confirm the presence of fats and oils.

### Quantitative phytochemical analysis

The identified phytochemicals were further quantified with a UV-vis spectrophotometer (Jenway 6305, Bibby Scientific Ltd., UK) using the methods of Harborne [23] and Trease and Evans [24]. The quantities of each phytochemical were extrapolated from the standard curve of their respective standard compounds, and the results were expressed in mg/l00 g.

**Test for steroids**; Each extract (1 g) was macerated with 20 mL of ethanol (Sigma Cat. No. 108543) and filtered. Subsequently, 2 mL of chromagen solution was added to the filtrate (2 mL), and the solution was left to stand for 30 min before measuring the absorbance at 550 nm. The concentration of steroids was extrapolated from the testosterone standard curve.

**Test for tannins**: Each extract (1 g) was macerated with 50 mL of methanol and filtered. Then, 0.3 mL of 0.1 N ferric chloride in 0.1 N HCl and 0.3 mL of 0.0008 M potassium ferricyanide were added to 5 mL of the filtrate, and the absorbance was read at 720 nm. The tannin content was extrapolated from a tannic acid standard curve.

**Test for soluble carbohydrates**: Each extract (1 g) was macerated with 50 mL of distilled water and filtered. Afterwards, a saturated aqueous solution of picric acid (Sigma Cat. No. 197378) was added to 1 mL of the filtrate, and the absorbance of the resulting solution was measured at 580 nm. The soluble starch standard curve was used to extrapolate the concentration of soluble carbohydrates in each extract.

**Test for cyanogenic glycosides**: Alkaline picrate (4 mL) was added to 1 g of each extract, and the mixture was shaken thoroughly before incubating in a water bath at 30 °C. Subsequently, the absorbance was read at 490 nm. Cyanogenic glycoside content was extrapolated from the KCN standard curve.

**Test for saponins**: A quantity of 0.5 g of each extract was macerated in 10 mL of petroleum ether (Sigma Cat. No. 101769). Then, another 10 mL of petroleum ether was added to the same beaker, and the filtrate evaporated to dryness. The residue formed was dissolved in ethanol (6 mL). Chromagen solution was added to 2 mL of the solution before incubating it for 30 min. at room temperature. The absorbance was measured at a wavelength of 550 nm. The contents of saponins in the extracts were calculated from the standard graph of diosgenin.

**Test for reducing sugars**: Each extract (0.5 g) was macerated with 20 mL of distilled water and filtered. 1 mL of the filtrate was added to 1 mL of alkaline copper reagent. The mixture was boiled for 5 min and cooled. Then, 2 mL of distilled water and 1 mL of phosphomolybdic acid reagent (Sigma Cat. No. 221856) were added, and the absorbance was measured at a wavelength of 420 nm. A standard glucose curve was used to extrapolate the quantity of reducing sugar in each extract.

**Test for glycosides:** A quantity (0.5 g) of each extract was macerated with 50 mL of distilled water and filtered. Afterwards, 4 mL of alkaline pirate solution was added to the filtrate (1 mL). The mixture obtained was boiled for 5 min and allowed to cool. The absorbance was read at a wavelength of 490 nm, and the quantity of glycosides in the extracts was obtained from the linalool standard curve.

**Test for flavonoids:** Each extract (1 g) was macerated with 20 mL of ethyl acetate for 5 min and filtered. To the filtrate (5 mL), 5 mL of dilute ammonia was added and shaken for 5 min. The upper layer was collected, and the absorbance was read at 490 nm. The amount of flavonoids in each extract was extrapolated from the quercetin standard curve.

**Test for alkaloids**: Each extract (1 g) was macerated with 20 mL of ethanol and 20% sulfuric acid (1:1 v/v). The filtrate (1 mL) was added to 5 ml of 60% sulfuric acid. After 5 min, 5 mL of 0.5% formaldehyde in 60% sulfuric acid was added to the mixture and allowed to stand for 3 h. The absorbance was read at 565 nm. The standard curve of caffeine was used to calculate the alkaloid content of each extract.

**Test for terpenoids**: Each extract (1 g) was macerated with 50 mL of ethanol and filtered. Then 2.5 mL of a 5% aqueous phosphomolybdic acid solution was added to the filtrate (2.5 mL), followed by the gradual addition of 2.5 mL of conc. Sulfuric acid. The mixture obtained was left to stand for 30 min. and then made up to 12.5 mL with ethanol. The absorbance was taken at 700 nm. The terpenoid standard curve was used to extrapolate the terpenoid quantity in each extract.

### Acute toxicity and lethality

Extracts’ acute toxicity and lethality were investigated using Lorke’s [25] method in two phases using 36 Swiss albino mice. The LD_50_ was calculated using the following equation:

$${\textrm{LD}}_{50}=\sqrt{Do}\times \surd D100.$$

Do and D100 are the maximum doses of the extracts that caused no mortality and the minimum dose that caused mortality, respectively.

### Selection of treatment doses

The doses of the extracts used for the study were chosen within the range of concentrations that gave optimal activity in the preliminary analysis. The acute toxicity test results showed that these doses were safe because they were lower than the maximally tolerated doses. However, the doses of the conventional drugs were translated from the human dose for each drug using the human equivalent dose conversion formula. Accurately weighed quantities of each extract and standard drug were suspended in Tween 80 to prepare suitable dosages.

### Assay of in vivo leukocyte mobilization

The experimental procedure of Ribeiro et al. [26] was used to investigate the effect of the extracts on in vivo leukocyte migration. Albino rats (40) were randomly distributed into eight experimental groups (*n* = 5). Group 1 served as the control (untreated, given 0.3 mL of the vehicle, tween 80); groups 2–8 were pretreated orally with levamisole (25 mg/kg b.w.) for group 2; 100, 250, and 500 mg/kg LMPGE for groups 3–5, respectively; and groups 6–8 were administered 100, 250, and 500 mg/kg LMPBE, respectively. The fluid obtained from the rat’s peritoneum was washed twice with heparinized (5 U/mL) phosphate-buffered saline (hPBS), and total leucocyte counts (TLC) were determined in the Neubauer chamber. For differential leucocyte counting (DLC), a cell pellet was obtained by centrifugation and resuspended in 1 mL of hPBS containing 5% serum albumin. The cells were stained with Wright’s stain, and the DLC was counted under light microscopy. The results were reported as the number of cells per cavity (Souza and Ferreira 1985).

### Delayed-type hypersensitivity (DTH) response

The Naved et al. [27] procedure was employed to investigate the DTH response. The 40 albino rats used in the study were divided into eight groups, as reported above. The treatment was done 3 days before sensitization on day 0 with 0.02 mL of 109 cells/mL of antigen (SRBCs) and continued daily until challenge on day 5. The difference in paw size before and 24 h after the challenge was used to calculate the DTH response.

### Hemagglutination antibody (HA) titre

The effects of the extracts on the humoral immune response were studied using the Sharma et al. [28] hemagglutination antibody (HA) titre assay. The antibody titer was expressed in a graded manner, with the minimum dilution (1/2) calculated as Log2 of the dilution factor being ranked 1.

### Cyclophosphamide-induced myelosuppression in rats

The Rasheed et al. [20] experimental procedure, with minor modifications, was used to assess cyclophosphamide-induced myelosuppression in rats. Myelosuppression was induced by intraperitoneal injection (i.p.) of cyclophosphamide (30 mg/kg b.w.). The rat total leukocyte counts (TLC) were determined on day 0 before suppression (TLC-A) and the seventh post-treatment day (TLC-B).

## Statistical analysis

The generated data sets were analyzed with IBM Statistical Package for Service Solutions (SPSS) version 23 using one-way analysis of variance (ANOVA). In addition, Duncan’s multiple comparisons and the T-test were employed to compare the significant differences between the groups at *p* <  0.05. The results are presented as the mean ± standard deviation (SD) of replicated values.

## Results

### Qualitative and quantitative phytochemical composition of LMPGE and LMPBE

From the qualitative and quantitative phytochemical analysis, both extracts (LMPGE and LMPBE) contain steroids, tannins, soluble carbohydrates, cyanogenic glycosides, saponins, reducing sugars, glycosides, flavonoids, alkaloids, and terpenoids, while resins, fats, and oils were absent (Table 1). The LMPGE was found to contain significantly (*p* <  0.05) more amounts of tannins, cyanogenic glycosides, saponins, reducing sugars, glycosides, flavonoids, and alkaloids than LMPBE (Table 1). However, soluble carbohydrate was significantly (*p* <  0.05) more abundant in LMPBE than LMPGE.

**Table 1:** Qualitative and quantitative phytochemical composition of LMPGE and LMPBE

S/N	Phyto-chemicals	Qualitative analysis				Quantitative analysis		
		Assay	Observation	LMPGE	LMPBE	LMPGE (mg/100 g)	LMPBE (mg/100 g)	*p* -values
1	Steroid	Cone. H_2_SO_4_	Reddish brown colouration	+	+	4.44 + 0.003^a^	4.43 + 0.005^a^	0.1971
2	Tannin	Braymer’s test	Greenish brown precipitate	+	+	2.87 + 0.003^b^	1.97 + 0.003^a^	0.0163
3	Soluble carbohydrates	Molish’s test	Purple interfacial ring	+	+	0.96 + 0.004 ^a^	1.43 + 0.002 ^b^	< 0.0001
4	Cyanogenic glycoside		Brown ring	+	+	0.07 + 0.004^b^	0.05 + 0.002 ^a^	0.0006
5	Saponins	Frothing’s test	Persistence foaming	+	+	2.03 + 0.004^b^	1.22 + 0.002^a^	< 0.0001
6	Reducing sugars	Fehling’s test	Brick red precipitate	+	+	56.52 + 0.003^b^	43.49 + 0.003 ^a^	< 0.0001
7	Glycoside	Sulphuric acid test	Brick red precipitate	+	+	1.19 + 0.003^b^	0.99 + 0.002^a^	< 0.0001
8	Flavonoids	Ethylacetate test	Intense yellow colouration	+	+	5.36 + 0.003^b^	4.79 + 0.003^a^	0.0306
9	Alkaloids	Wagner’s test	Reddish-brown ppt	+	+	13.39 + 0.009^b^	11.35 + 0.002^a^	< 0.0001
10	Terpenoids		Grey coloration	+	+	0.49 + 0.003^a^	0.48 + 0.001^a^	0.8593
11	Resin	Acetic anhydride test	No orange-to-yellow coloration	–	–	–	–	–
12	Fats and oil	Spot test/ Stain test	No oil stain on the filter paper	–	–	–	–	–

The key, +, indicates “the presence or availability of phytochemical” while -, indicate their absence (Qualitative results). The data of the quantitative analysis represent the mean ± SD of triple determination. Values with different superscripts in a row are significantly different, while mean values with the same letter as a superscript are not significantly different at *p* < 0.05. The results were analyzed with ANOVA, and post hoc multiple comparisons of each mean value were done with the T-test

Quantitative values with different superscripts (a or b) in a row are significantly different, while mean values with the same letter (a) as a superscript are not significantly different at *p* < 0.05

### Acute toxicity test (LD_50_) of LMPGE and LMPBE

Both LMPGE and LMPBE showed no signs of acute toxicity in mice that received the extracts at doses from 10 to 2900 mg/kg b.w. However, administration of a higher dosage (5000 mg/kg b.w.) provoked drowsiness and weakness in the mice, although no death was recorded after 48 h (Table 2)

**Table 2:** The median lethal dose of the methanol extract of *Loranthus micranthus*

Dosages (mg/kg b. w.)	LMPGE		LMPBE	
	Mortality	BΔ	Mortality	BΔ
10	0/3	Nil	0/3	Nil
100	0/3	Nil	0/3	Nil
1000	0/3	Nil	0/3	Nil
1600	0/3	Nil	0/3	Nil
2900	0/3	Nil	0/3	Nil
5000	0/3	Drowsiness and weakness	0/3	Drowsiness and weakness

*n =* 3 mice in the groups, while BΔ stands for behavioural changes

### Effects of LMPGE and LMPBE on total and differential leukocyte counts

**Fig. 1:**
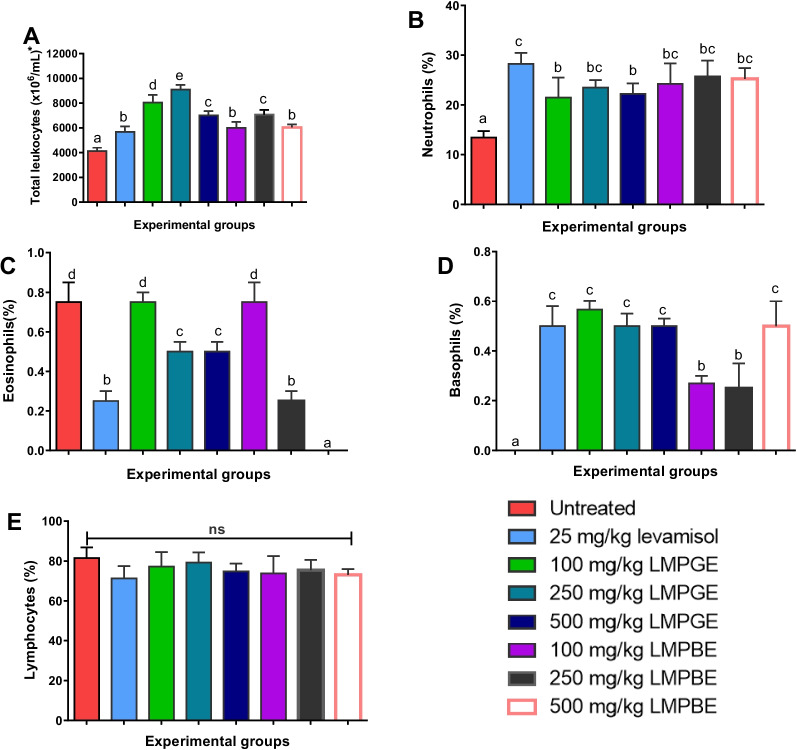
The effects of LMPGE and LMPBE on total and differential leukocyte counts

The rats treated with both LMPGE and LMPBE (experimental groups 3 to 8) prior to agar-induced in vivo leukocyte migration showed a significant (*P* < 0.05) increase in the total number of leucocytes as well as neutrophils and basophils when compared to the untreated group (group 1). Moreover, there were no significant differences (*p* > 0.05) in the lymphocyte count across the groups, and the number of eosinophils showed contrasting trends, as they decreased significantly in the some of treated groups compared to the untreated. Although the results did not follow a consistent dose-dependent pattern for all the immunological parameters examined (Fig. 1), it is interesting to note that both extracts (LMPGE and LMPBE) showed either significantly higher or similar outcomes compared to the experimental group (group 2) administered or treated with the standard drug (25 mg/kg b.w. of levamisole). Finally, from our results, LMPGE showed better immunostimulatory activities than the LMPBE, as they fostered a greater increase in total leucocyte count as well as basophil number in the experimental group administered 250 and 500 mg/kg.

The values are presented as the mean ± SD of five rats. Values with a different alphabet in a given parameter are statistically significant at *p* < 0.05. However, mean values with the same lettered alphabet are not significantly different (*p* < 0.05). The results were analyzed with ANOVA, and post hoc multiple comparisons of each mean value were done with Duncan’s multiple tests.

### Effects of LMPGE and LMPBE on delayed-type hypersensitivity induced by SRBCs

**Fig. 2:**
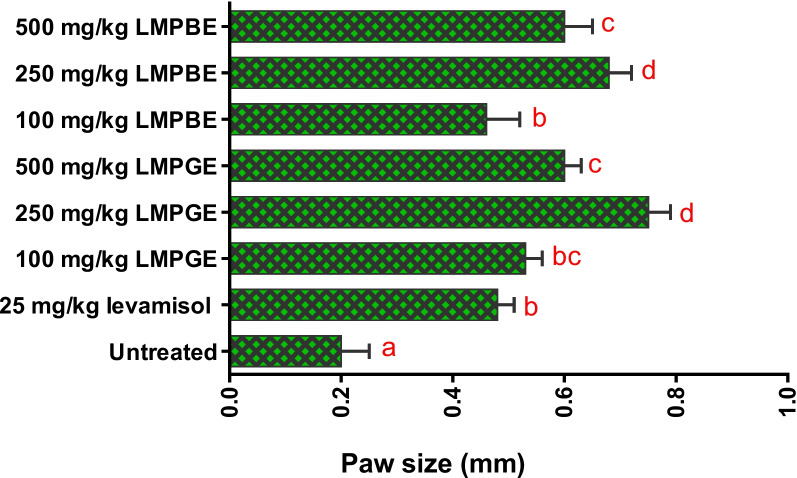
Effects of LMPGE and LMPBE on delayed-type hypersensitivity induced by SRBCs

Oral treatment with LMPGE and LMPBE significantly (*P* 0 < .05) increased the paw sizes of the rats in a non-dose-dependent manner as compared to the untreated group. Interestingly, it was observed that the groups treated with 250 mg/kg b.w. of both extracts had larger paw size than what was recorded in levamisole-treated rats (Fig**.** 2).

The values are presented as the mean ± SD of five rats. Values with different alphabets are statistically significant at *p* < 0.05. However, mean values with the same lettered alphabet are not significantly different (*p* < 0.05). The results were analyzed with ANOVA, and post hoc multiple comparisons of each mean value were done with Duncan’s multiple tests.

### Effects of LMPGE and LMPBE on hemagglutination antibody (HA) titers

**Fig. 3:**
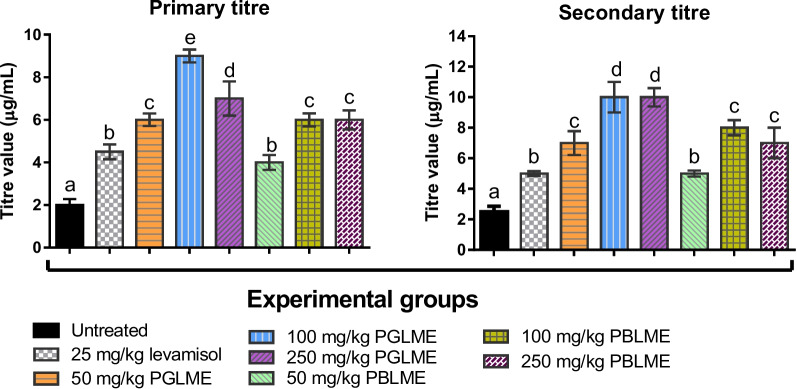
Primary and secondary antibody titres of rats challenged with SRBCs

As shown in Fig**.** 3, groups treated with the extracts after SRBC immunization and challenge had significant (*p* < 0.05) elavation in primary and secondary antibody titers relative to the negative control (group 1). The increase in the primary titre values was highest in groups 4 and 5 treated with 100 and 250 mg/kg b.w. of LMPGE, respectively. Moreso, the primary and secondary antibody titers of the extract-treated groups were found to be significantly (*p* < 0.05) higher than those of group 2, treated with levamisole, with the exception of the group administered 50 mg/kg b.w. of LMPBE.

The values are presented as the mean ± SD of five rats. Values with a different alphabet in a given parameter are statistically significant at *p* < 0.05. However, mean values with the same lettered alphabet are not significantly different (p < 0.05). The results were analyzed with ANOVA, and post hoc multiple comparisons of each mean value were done with Duncan’s multiple tests.

### Effects of LMPGE and LMPBE on cyclophosphamide-induced myelosuppression

The rats treated with both LMPGE and LMPBE extracts (experimental groups 3 to 8) after cyclophosphamide induction showed a significant (*p* < 0.05) dose-dependent increase in the TLC, with LMPGE having a higher potency**.** Furthermore, it was also observed that the extracts increased TLC more than levamisole, except for the group treated with 50 mg/kg b.w. of LMPBE (Table 3).

**Table 3:** Effects of LMPGE and LMPBE on cyclophosphamide-induced myelosuppression

Experimental groups	Treatments (mg/kg b.w.)	TLC-A (× 10^9^ /L)	TLC-B (× 10^9^ /L)
Negative control	–	11,950.00 ± 30.00	3425.00 ± 25.00
Levamisole	25	11,000 ± 23.17	5212.50 ± 10.00*
LMPGE	50	12,110 ± 17.29	6200.00 ± 30.00*
	100	12,200 ± 15.01	7812.50 ± 192.00*
	250	1275 ± 15.00	9712.50 ± 178.00*
LMPBE	50	11,950 ± 47.00	5225.10 ± 195.00*
	100	12,175 ± 132.00	7137.50 ± 165.00*
	250	11,475 ± 170.00	8000.00 ± 105.00*

TLC-B vValues with asterisks (*) differed significantly at *p* < 0.05 compared with the untreated group (negative control)

The values are presented as the mean ± SD of five rats. TLC A and TLC B stand for TLC before suppression and after suppression, respectively. Values with asterisks differed significantly at *p* < 0.05 compared with the untreated group (negative control). The results were analyzed with ANOVA, and post hoc multiple comparisons of each mean value were done with Duncan’s multiple tests.

## Discussion

The immunomodulatory activity of natural products that stimulate or suppress the immune system offers excellent therapeutic benefits in regulating immune-mediated diseases [1, 6]. In the current study, the phytochemical screening showed that the extracts are rich in reducing sugars, alkaloids, flavonoids, steroids, and tannins, whereas saponins were present in low amounts. These results agree with the work of Govindappa et al. [29], who also recorded a high abundance of alkaloids, flavonoids, phenols, and tannins in methanol extracts of *L. micranthus* growing on *Azadirachta indica* except that saponins were not identified from their n-hexane and methanol extracts. The possible reason for this slight difference in the phytochemical composition of the plants collected from different hosts and locations is supported by the fact that the phytochemical contents are influenced by the host plant, habitat, and environmental factors [30]. According to ethnopharmacological studies, these identified phytoconstituents have sound therapeutic effects [31–33]. Among the identified phytochemicals, saponins have cytotoxic, antiallergic, antiviral, molluscicidal, and immunomodulating activities [34]. Flavonoids and tannin compounds contain multiple hydroxyl groups required to neutralize free radicals, chelate metal ions, decompose peroxides, and quench singlet oxygen [35]. According to Yahfouf et al. [36], polyphenols regulate the immune system through their regulatory actions on signaling pathways that are not limited to the mitogen-activated protein kinases (MAPk), Janus kinases-signal transducer and activator of transcription proteins (JAK/STAT), nuclear factor-κB (NF-κB), and arachidonic acid pathways. Notably, the high amounts of alkaloids in the extracts evoke much research interest due to their biological activity and therapeutic potential. Alkaloids have anticancer, antioxidant, antimicrobial, anti-inflammatory, and immunomodulatory properties [32, 37]. Collectively, the presence of these phytochemicals with immune modulatory effects suggests that the extracts could have promising potential in treating immune-related diseases.

Medicinal plants are considered an alternative therapy against the severe adverse effects of synthetic immunomodulatory drugs [38]. However, there is no inclusive technical information regarding their safety [2]. Undoubtedly, toxicological evaluation of medicinal plants is necessary to ascertain their safety for human use [22]. The acute toxicity study results of the extracts showed no observable signs of toxicity or death after 48 h of administration of 2900 mg/kg b.w. of both extracts. The drowsiness and weakness observed after oral administration of 5000 mg/kg b.w. show that *L. micranthus* should not be administered at this dose to avert these symptoms. According to Porwal et al. [39], scientific knowledge of acute toxicity is vital to identify doses that could be used in animal studies and clinical trials and to reveal any possible clinical signs provoked by drugs under investigation. The safety profile of *L. micranthus* has previously been reported by Ani et al. [40], who did not record any organ damage in the histological architecture of major organs after sub-acute toxicity studies in animals administered *L. micranthus* extract. Likewise, our result corroborated that of Jimoh et al. [41], who did not identify any adverse biochemical changes or organ toxicity after mistletoe leaf meal-supplemented diets. Based on the acute toxicity test findings, the extracts were considered suitable for usage as medicine, and all these results above served as a basis for choosing the actual dose selection for in vivo immunological studies.

Leukocyte migration is paramount for transporting cellular components of the immune system to eliminate pathogenic stimuli. The decrease in TLC and DLC, major biomarkers of immune function, in the negative control group could be a pointer to a poor immune response. Interestingly, although the lymphocyte counts remained relatively constant, we recorded an increase in TLC and the number of immune cells, such as neutrophils and basophils. The increment in TLC and DLC in the extracts-treated groups in a manner comparable with the levamisole-treated groups (positive control) suggests possible immune stimulation. This finding agrees with those of Ogbue et al. [42] and Dogan [43], who also recorded an increase in leucocyte counts after treatment with *Musanga cecropioides* and levamisole, respectively. Dogan [43] hypothesized that the imidazole and sulfur structures of levamisole have stimulant effects on T lymphocytes. Remarkably, empirical studies have reported that pharmaceutical agents that enhance the TLC and DLC could lower the risk of opportunistic infections and avert the risk of mortality and morbidity due to compromised immune status [42]. The reported immune-enhancing effects of the extracts may be underpinned by the high abundance of polyphenols, especially flavonoids and tannins, already known as active immunostimulatory agents [42]. As Yahfouf et al. [36] described, polyphenols regulate the immune system by increasing the activity of T-cells, interferons (IFNs), cytokines, macrophages, and B-lymphocytes. Notably, the synergistic effects of these phytochemicals might have elicited the mobilization of leucocytes, which are an integral part of cell-mediated and humoral immunity [11], thereby unveiling the immunomodulatory action of the extracts.

The DTH response is an antigenic-cell-mediated immune-inflammatory reaction modulated by T lymphocytes, macrophages, and their products [44]. From literature, antigens sensitize T cells to release proinflammatory mediators leading to increased DTH reaction in animals, as shown by paw edema [45]. Thus, we investigated the paw sizes of the SRBC-induced antigenic rats to establish a scientific basis for developing immune inflammation. According to our findings, treatment with the extracts significantly (*p* < 0.05) increased the paw size of the SRBC-induced antigenic rats compared with the negative control, with maximal effects recorded at 250 mg/kg b.w. Currently, we cannot pinpoint the precise cause of the 500 mg/kg body weight dose-related decrease in effect. Nevertheless, the reduced effect may have resulted from an inhibition brought on by an excessive dosage of each extract present at the site of action. Furthermore, the paw size diminution at 500 mg/kg b.w. might indicate that the extracts also exhibit anti-inflammatory properties at that dosage. This is another possible thrust for future research with the extracts. Remarkably, the decrease in paw size at the higher doses is consistent with the findings of Osadebe and Omeje [21], who also obtained a reduction in the DTH response at higher doses of Nigerian mistletoe epiphytic on *Kola acuminate, Persia americana,* and *Pentaclathra macrophylla*. This is a pointer that the extracts should be administered at low concentrations to achieve optimal immune stimulatory effects. Interestingly, our findings corroborate Pollard and Bijker’s hypothesis [44] that immunostimulants such as vaccines must be administered at relatively low doses to achieve optimal effects. The fact that the positive control increased the paw volume corresponds with former reports that levamisole increases the number of T cells by stimulating phagocytes, enhancing sensitivity to antigens and mitogens [43]. Moreover, the increase in paw size after treatment with the extracts suggests that the extracts may activate adaptive immune responses since research studies have established that an increment in DTH reaction in the SRBC-induced antigenic rats after treatment shows activation of adaptive immunity [3, 20]. This potential might be through the increased secretion and activation of lymphocytes and pro-inflammatory mediators involved in the DTH reaction [20]. Generally, the activated cytokines and mediators increase vascular permeability, exuding immune cells and activating macrophage cells in the challenged region, which prompt phagocytosis and successfully eliminate the antigen or infection [30]. Imperatively, the increase in paw size by the extracts may be attributed to the immune-stimulating effects of its active phytoconsituents.

Antibody production involves several immune actions, such as antigen processing, presentation, antigen recognition by pathogen-recognition receptors (PRRs), and cytokine activation of the memory B-cell response [46]. Thus, measuring primary and secondary antibody titers is valuable for evaluating humoral immunity. An increase in primary and secondary antibody titers was recorded in groups treated with levamisole and graded doses of the extracts, with LMPGE having higher potency. This result is consistent with those of El-Sawy et al. [47], who discovered that levamisole supplementation provoked a marked increase in antibody titer against Newcastle disease. The increased antibody titre in the treated groups might be due to the mobilized leucocytes, which have stimulatory effects on B and T cells involved in antibody production. At the same time, the increase in the secondary titre connotes the presence of memory cells. This result supports the hypothesis that subsequent antigenic stimulation enhances the responsiveness of macrophages and lymphocytes required for antibody synthesis because there is now an expanded clone of cells with a memory of the original antigen available to proliferate into the mature cell [48]. Previous studies have reported that an increase in antibody titre increases humoral response, preventing disease progression and microbial dissemination and enhancing microbial clearance [42]. The reported increase in antibody titre by the extracts might be due to the synergistic effects of their phytochemicals, especially flavonoids and tannins, already known as active immunostimulatory agents. Behl et al. [37] pointed out that these phytochemicals modulate signaling pathways involved in cytokine synthesis, enhance antigen recognition, and stimulate immune cells. The abundance of these phytochemicals in LMPGE may account for its higher immunomodulatory action. This concurs with the findings of Shabbir et al. [49], who reported high immunomodulatory effects of *Psidium guajava*. Thus, the difference in the humoral immunity of *L. micranthus* extracts collected from the two host trees supports the possibility of metabolic exchange between the mistletoe and the host tree. These findings agree with what has been established in several studies in literature: that several factors, including the host and species of mistletoe, play essential roles in the pharmacological activities of mistletoe [16, 29].

Cyclophosphamide (CP) induces immunosuppression by interfering with DNA and RNA functions, inhibiting DNA synthesis, and cross-linking DNA, ultimately hindering B cell proliferation and lymphocyte proliferation [50]. To investigate the effects of the extracts on the immunosuppressed state, we generated a myelosuppressive rat model using CP. We discovered that, although CP administration decreased the TLC, consistent with prior reports by Parveen et al. [6] and Noh et al. [11], treatment with both extracts significantly (*p* < 0.05) increased TLC in a dose-dependent manner. These results suggest that the extracts could attenuate the myelosuppressive effects of CP. Restoration of the TLC in the treated rats, which was more pronounced in LMPGE, may be due to its higher phytochemical contents. Previously, studies have reported that phytochemicals exert complementary and synergistic effects to increase the release and activation of immune cells and mediators involved in activating cell-mediated and humoral immune responses [6]. The increase in TLC is a pointer to the protective effects of the extracts against CP-induced suppression of T and B cell proliferation. Studies have shown that an increase in TLC increases host defense against the inversion of foreign agents and is also a possible means of inhibiting disease progression in humans without eliciting harmful effects [51]. Thus, the extracts may be helpful as immune stimulants.

## Conclusions

Our preliminary experiments identified a high abundance of pharmacologically relevant phytochemicals, which were higher in LMPGE extract. Furthermore, this study provides suggestive clues towards the immunomodulatory potential of the extracts, as evidenced by activation in leucocyte mobilisation, paw odema, antibody titre values, and TLC in rats after immune suppression. However, we recommend further studies regarding the characterization, isolation, and elucidation of the detailed molecular mechanism(s) responsible for the immunomodulatory potential of the extracts to fully harness their health-promoting properties.

## Data Availability

All data generated or analyzed during this study are included in this published article.

## Abbreviations

Ab: Antibody
CP: Cyclophosphamide
DTH: Delayed-type hypersensitivity
DLC: Differential leukocyte counts
HA: Hemagglutination antibody
Ig: Immunoglobulin;
IFN: Interferon
IL-4: interleukins-4
JAK/STAT: Janus kinases -signal transducer and activator of transcription proteins
LD_50_: Lethal Dose
*L. micranthus*: *Loranthus micranthus*
LMPBE: *L. micranthus* epiphytic on *Parkia biglobosa*
LMPGE: *L. micranthus* extracts epiphytic on *Psidium guajava*
MAPk: Mitogen-activated protein kinases
NF-κB: Nuclear factor-κB
SRBCs: Sheep red blood cells
TLC: Total leukocyte counts
TNF-α: tumour necrosis factor-α.
